# Alternative splicing, FOXP3 and cardiovascular disease

**DOI:** 10.18632/aging.101897

**Published:** 2019-04-12

**Authors:** Anne-Laure Joly, John Andersson

**Affiliations:** 1 Karolinska Institutet, Stockholm 17177, Sweden

**Keywords:** FOXP3, isoforms, atherosclerosis

Atherosclerosis is a chronic inflammatory disorder of large- and medium-sized arteries and remains one of the most common causes of age-related death worldwide. It is well established that effector T cell responses promote both atherosclerotic plaque growth and instability [1]. In contrast, a number of studies have demonstrated a protective effect of immunosuppressive CD4+FOXP3+ regulatory T (Treg) cells in experimental models of atherosclerosis [1]. This has led to substantial efforts in developing Treg cells based therapeutic approaches including vaccines. One major challenge for translating these experimental findings into a clinical setting is that not all aspects of atherosclerotic disease nor Treg cells are conserved across species. One biological process that frequently generates such species differences is alternative splicing. Alternative splicing is a carefully regulated process that allows the generation multiple protein products from one single gene [2]. This is normally accomplished by selected exclusion of defined exons, leading to multiple mRNA transcripts. Importantly the resulting proteins may have fundamentally different roles, which is exemplified by the genes in the P53 family where alternative splicing gives rise to dominant negative isoforms [3]. Alternative splicing occurs in approximately 95% of all human genes and 60% of all mouse genes. As a consequence, alternative splicing adds to the complexity when translating findings in experimental systems to the clinics.

Treg cells are a potential mediator of future intervention strategies for atherosclerosis. These cells depend on the lineage-defining transcription factor FOXP3 for their development and function. Human FOXP3 isoforms are generated through the exclusion of exon 2 and/or exon 7. In contrast, mice do not produce any isoforms of FOXP3. Full-length FOXP3 (FOXP3fl) and FOXP3 lacking exon 2 (FOXP3Δ2) isoforms confer a suppressive ability to Treg cells *in vitro* [4–6], whereas FOXP3 lacking exons 2 and 7 (FOXP3Δ2Δ7) has been suggested to inhibit FOXP3fl and FOXP3Δ2 in a dominant-negative manner [7]. The differential functions of FOXP3 isoforms throws a wrench into the interpretation of many previous studies trying to correlate total FOXP3 expression with disease progression/severity in the clinics. This is illustrated by studies of FOXP3 isoforms in chronic inflammatory disorders such as Crohn’s disease and atherosclerosis. Here we have been unable to correlate the degree of total FOXP3 expression with disease progression. However, an increased disease severity in Crohn’s disease is associated with alternative splicing of *FOXP3* exon 7, whereas, a reduced portion of FOXP3Δ2 is associated with an unstable plaque phenotype [6,7]. Thus, alternative splicing of *FOXP3* appears to represent a layer of regulation that can reveal functional differences of Treg cell populations in different patient categories.

The two dominant FOXP3 isoforms, FOXP3fl and FOXP3Δ2, are coexpressed in Treg cells and normally makes up 95% of the total amount of FOXP3 protein. The expression pattern does however vary. For instance, we have found that IL-1β could promote splicing of *FOXP3* exon 7 [7]. Recently, we also found that TCR activation leads to specific upregulation of FOXP3Δ2 [6]. Another recent study suggested that FOXP3fl instead increased upon TCR stimulation [8]. However, a confounding factor of this study is that the authors assessed FOXP3 isoform composition in cultured CD4+ effector T cells rather than in defined populations of Treg cells and that the data is based only on flow cytometry analysis [8]. As we have shown, analysis using flow cytometry, western blot and quantitative PCR gives a more accurate estimation of FOXP3 isoforms [7]. Importantly, our findings imply that the composition of FOXP3 isoforms modulates the phenotype of Treg cells upon environmental cues. They also reinforce the notion that it is crucial to define the FOXP3 isoform composition when studying Treg cells in clinical samples.

What will the future bring to FOXP3 isoforms and their involvement in chronic inflammatory disorders? First, we hope that the studies involving FOXP3 isoforms will be expanded upon as it remains essential to validate the current findings in other patient cohorts. This should go hand in hand with developing better tools for identification of specific FOXP3 isoforms. Another interesting line of research will be to better define the molecular mechanisms of action of distinct FOXP3 isoforms including their interactomes and their differential ability to control gene expression. Finally, we have together with collaborators, identified patients with atypical forms of the immunodysregulation polyendocrinopathy enteropathy X-linked syndrome that lack expression of specific FOXP3 isoforms. We believe that these patients will give us a better understanding of the *in vivo* function of FOXP3 isoforms in humans.
